# Radio-Protective Effects of Stigmasterol on Wheat (*Triticum aestivum* L.) Plants

**DOI:** 10.3390/antiox11061144

**Published:** 2022-06-10

**Authors:** Hebat-Allah A. Hussein, Shifaa O. Alshammari, Fatma M. Elkady, Amany A. Ramadan, Sahar K. M. Kenawy, Aisha M. Abdelkawy

**Affiliations:** 1 Botany and Microbiology Department, Faculty of Science (Girls Branch), Al-Azhar University, Cairo 11754, Egypt; saharkenawy.2052@azhar.edu.eg (S.K.M.K.); aisha.abdelgalil.5921@azhar.edu.eg (A.M.A.); 2 Biology Department, University College of Nairiyah, University of Hafr Al-Batin, Nairiyah 31991, Saudi Arabia; 3 Biology Department, College of Science, University of Hafr Al-Batin, Hafr Al-Batin 39524, Saudi Arabia; dr.shifaa@uhb.edu.sa; 4 Department of Botany, Agriculture, Biological Research Institute, National Research Centre, Dokki, Giza 12311, Egypt; fatma_elkady99@yahoo.com (F.M.E.); amanyramadan66@yahoo.com (A.A.R.)

**Keywords:** antioxidants, biochemical metabolites, plant growth, stigmasterol, phytohormones, wheat

## Abstract

Ionizing radiation is abiotic stress limiting the growth and productivity of crop plants. Stigmasterol has positive effects on the plant growth of many crops. The role of stigmasterol in alleviating the effects of ionizing radiation on plant metabolism and development is still unclear. Therefore, the study aimed to investigate the effects of pretreatments with γ-radiation (0, 25, and 50 Gy), foliar application of stigmasterol (0, 100, and 200 ppm), and their interaction on the growth, and biochemical constituents of wheat (*Triticum aestivum* L., var. Sids 12) plants. Gamma radiation at 25 Gy showed no significant difference in plant height, root length, no. of leaves, shoot fresh weight, root fresh weight, Chl *a*, ABA, soluble phenols, and MDA compared to the control values. Gamma rays at 50 Gy inhibited shoot and root lengths, flag leaf area, shoot fresh and dry weights, photosynthetic pigments, total soluble sugars, proline, and peroxidase activity. However, it stimulated total phenols, catalase activity, and lipid peroxidation. On the other hand, stigmasterol at 100 ppm showed no significant effects on some of the physiological attributes compared to control plants. Stigmasterol at 200 ppm improved plant growth parameters, photosynthetic pigments, proline, phenols, antioxidant enzyme, gibberellic acid, and indole acetic acid. Correspondingly, it inhibited total soluble sugars, abscisic acid, and lipid peroxidation. Moreover, the application of stigmasterol caused the appearance of new polypeptides and the reappearance of those missed by gamma radiation. Overall, stigmasterol could alleviate the adverse effects of gamma radiation on wheat plants.

## 1. Introduction

Wheat (*Triticum aestivum*, L.) is the main strategic cereal crop. It has high nutritional value as it is rich in carbohydrates, essential amino acids, fiber components, vitamins, and minerals [1]. Global wheat production amounted to around 778.6 million tons from 2020 to 2021 [2]. Wheat is used for making bread, starch, and wheat germ oil. The nutritional value of grains of bread-making quality depends mainly on the various protein constituents [3]. Wheat plants are exposed to numerous stressors, such as ionizing radiation stress, that seriously affect plant growth and productivity [4].

Ionizing radiation is a pollutant that could potentially lead to disturbances in ecosystems [5]. Gamma-radiation is an electromagnetic wave that results in ionizing radiation that impacts different biological macromolecules and induces variable biological effects [6,7]. The effects of gamma-rays on plant growth and development are diverse, ranging from stimulatory to inhibitory effects depending on the radiation dose, exposure duration, the response and sensitivity of different plant species and cultivars, and their interaction inside the cell, especially in water, to produce free radicals [8] that change the biochemical processes of plants [9]. Previous studies have investigated the effect of dose and exposure time of gamma-ray irradiation on seed germination and physiological parameters. Gamma rays at 100 Gy enhanced growth, yield characters, and certain biochemical constitutes of fenugreek [10] and wheat plants [11]. A recent study revealed that IS-Jarissa wheat varieties were able to live after exposure to radiation doses of 0 Gy, 100 Gy, 200 Gy, 300 Gy, and 400 Gy [12]. Additionally, gamma radiation at 200 Gy enhanced flavonoid compounds in 37 wheat lines [13], while ionizing radiation from 10 to 1000 Gy caused dramatic alterations in the composition of plant cells and induced cell death [14,15]. Mean germination time, root and shoot length, and seedling dry weight of wheat genotypes (Roshan and T-65-58-8) decreased with increasing radiation doses (100, 200, 300, and 400 Gy) [16]. Additionally, the radiation doses of 10, 15, 20, 25 and 30 kR caused different types of chromosomal anomalies in wheat plants, which increased with the increasing intensity of gamma radiation [17].

Plants produce mixtures of sterols, including stigmasterol, campesterol, and sitosterol. Stigmasterol belongs to the plant sterols and is a precursor of brassinosteroids, which act as growth regulators [18]. Additionally, Campesterol is the precursor of BR; the crucial role of BR in plant growth and development is well established [19], while sitosterol participates in cellulose synthesis [20]. Stigmasterol is implicated in the structure of phospholipid constituents, maintaining plasma membrane fluidity and permeability [19,21,22]. Moreover, foliar application of stigmasterol improved the growth characters, yield, anatomical structures, and percentage/composition of essential oil of basil plants [23]. Stigmasterol may be involved in gravitropism and tolerance to abiotic stress [19]. Indeed, stigmasterol application of germinating seeds enhanced the salt tolerance of faba beans and flax plants [24,25]. It is, however, unclear whether stigmasterol can overcome the harmful effects of ionizing radiation stress. As the cost of stigmasterol is brought down to affordable levels [24], the present research theme may contribute greatly to the usage of stigmasterol in agriculture production as well as to overcome the threat of ionizing radiation on crop plants around the world. Therefore, our purpose is to examine the role of stigmasterol in alleviating the adverse effects of γ-radiation on wheat plants and to understand the direct role of stigmasterol in plant growth and stress responses.

## 2. Materials and Methods

### 2.1. Experimental Design

A greenhouse experiment was conducted during the winter season of 2017/2018 at the Faculty of Science (Girls Branch), Al-Azhar University (latitude 30°03′22.3″ N longitude 31°19′25.4″ E), Nasr City, Cairo, Egypt. The study aimed to test the effects of pre-treatment with γ-radiation (0, 25, and 50 Gy) and foliar application of stigmasterol (0, 100, and 200 ppm) on growth, biochemical constituents, and yield characters of wheat plants (*Triticum aestivum*, L.). Wheat grains (Cultivar Sids 12) were received from the Agriculture Research Center (latitude 30°01′13.4″ N longitude 31°12′24.2″ E), Giza, Egypt. Stigmasterol, purity ≥ 95, soluble in chloroform, 50 mg/mL, MP Biomedicals, LLC, France, was used. The seeds were irradiated by gamma ^60^Co at different doses including 0 (non-irradiated), 25, and 50 Gray (Gy) at the Egyptian Atomic Energy Authority (latitude 30°02′41.8″ N longitude 31°20′41.0″ E), Cairo, Egypt. Wheat grains were sown on November 21 in earthenware pots (no. 50) filled with sandy soil with six replicates for each treatment. The soil texture was sandy, field capacity 11.5%, pH 8.7, EC 0.35 dSm^−1^, Cl^−^ 1.7, HCO_3_^−^ 1.10, Na^+^ 1.2, K^+^ 0.25, Ca^2+^ 1.27%, and Mg^2+^ 0.58 meq L^−1^. Phosphorus fertilizer was added before sowing at a rate of 6.0 g per pot of calcium superphosphate (15.5% P_2_O_5_). Nitrogen fertilizer was applied in two equal portions at a rate of 0.60 g/pot for each in the form of ammonium nitrate (33.5%N) at 30 and 60 days after planting. Potassium fertilizer was applied as a soil application at the rate of 2 g/pot in the form of potassium sulfate (48–52% K_2_O) 45 days after planting. The foliar spray of stigmasterol treatments was applied at the vegetative stage (45 days after sowing).

### 2.2. Growth Parameters 

At 65 days after sowing, three representative samples were collected from each treatment for detecting the tested growth traits (shoot length (cm), root length (cm), leaves (no. per plant, flag leaf area), as well as fresh and dry weights of shoot and root per plant).

### 2.3. Photosynthetic Pigments

According to the procedure in [26], photosynthetic pigments—chlorophyll-a (Chl *a*), chlorophyll-b (Chl *b*)—and total carotenoids in samples of wheat fresh leaves tissues using 85% acetone with 0.1 g fresh weight (FW) of wheat leaves after grinding in the solvent was measured. The homogenized samples were centrifuged at 3000 rpm, and the filtrate was topped up to 10 mL with acetone (85%). The absorbance was recorded at 663, 644, and 452 nm using a spectrophotometer (VEB, Carl-Zeiss-Promenade, Jena, Germany) using acetone as a blank. The concentration of the pigment fractions (Chl *a*, Chl *b*, and carotenoids) was accounted for as µg/mL using the following equations:

Chl *a* = [(10.3 × E663) − (0.918 × E644)] = µg mL^−1^ (1)

Chl *b* = [(19.7 × E644) − (3.870 × E663)] =µg mL^−1^ (2)

Carotenoids = (4.2 × E452) − [(0.0264 × Chl *a*) + (0.426 × Chl *b*)] = µg mL^−1^ (3)

The concentrations of chlorophylls and carotenoids were expressed as mg g^−1^ fresh weight (FW) of plant material. Pigment contents are represented as mg g^−1^ FW. 

### 2.4. Identification of Endogenous Hormones

A total of 10 g of fresh tissue per sample was homogenized with 80% (*v*/*v*) ethanol and stirred overnight at 4 °C. The extract was filtered through a Whatman filter, and the methanol was evaporated under a vacuum. The aqueous phase was adjusted to pH 2.5 with 1 N HCl, then partitioned with ethyl acetate (3 times), and finally passed through anhydrous sodium sulfate. After that, the ethyl acetate phase was evaporated under a vacuum. The dry residue containing acidic hormones (fraction I) was dissolved in 2.0 mL of methanol and stored in vials at 4 °C. The phytohormones (auxins, gibberellins, and abscisic acid) were determined by high-performance liquid chromatography (Shimadzu, Tokyo, Japan), isocratic UV analysis, and a reverse-phase C18 column (RP-C18 μ Bondapak, Waters). The column used included octadecylsilane (ODS) ultra-sphere particles (5 μm), and the mobile phases used were acetonitrile/water (26:74 *v*/*v*) at pH 4.00. The flow rate was 0.8 mL min^−1^, and detection was UV 208 nm. The standard solutions of the individual acids (auxins, gibberellins, and abscisic acid (Sigma, St. Louis, MO, 63178, USA)) were prepared in the mobile phase and chromatographed. All solvents were purchased from Aldrich (Munich, Germany). 

### 2.5. Total Soluble Sugars

Total soluble sugars (TSS) were estimated in dry flag leaves of the wheat plant by the anthrone technique [27]. The TSS was analyzed by reacting 0.1 mL of ethanol extract with 3.0 mL freshly prepared anthrone (150 mg anthrone (Aldrich Chemical Company Inc., Milwaukee, WI 53233, USA) + 100 mL 72% H_2_SO_4_) in a boiling water bath for 10 min. The cooled samples were read at 625 nm using a spectrophotometer (VEB, Carl-Zeiss-Promenade, Jena, Germany). Total soluble sugar was calculated using a standard curve of glucose.

### 2.6. Proline

Proline content was determined according to [28]. A total of 0.5 g of fresh leaves was extracted in 10 mL of aqueous sulfosalicylic acid (3%). A total of 2 mL of the extract was taken and mixed with 2 mL of acid ninhydrin reagent (Alpha, Mumbai, 400 002, India) and 2 mL of glacial acetic acid for one h at 100 °C. After cooling, 4 mL of toluene was added to the reaction mixture to extract the proline content. The absorbance was recorded using a spectrophotometer (VEB, Carl-Zeiss-Promenade, Jena, Germany) at 520 nm using toluene as a blank. Free proline was determined from the standard curve of *L*-proline. 

### 2.7. Total Phenolics

Total phenolic content in dry leaves was estimated by the method described by [29,30]. One milliliter of the extract was added to ten drops of concentrated HCl in a boiling water bath for ten min and cooled. Then 1 mL of Folin–Ciocalteau reagent and 1.5 mL of 14% sodium carbonate were added to the extract. The mixture was completed with distilled water up to 5 mL, shaken well, and then kept in a boiling water bath for five minutes. The absorbance at 650 nm was noted, and the data were represented as mg g^−1^ FW using a pyrogallol standard curve. 

### 2.8. Assay of Antioxidant Enzymes

The crude enzyme was obtained according to the assay of antioxidant enzyme activities. A fresh flag leaf (2 g) of wheat plants was extracted in 10 mL of 100 mM phosphate buffer (pH 6.8) and kept at 4 °C overnight. The extract was centrifuged at 5000 rpm for ten minutes and reserved to assay the activities of enzymes [31]. 

#### 2.8.1. Peroxidase (POX) Assay 

POX activity was assayed according to [32]. A total of 0.2 mL of crude extract was reacted with 5.8 mL of phosphate buffer (50 mM, pH 7.0), 2.0 mL pyrogallol (20 mM), and 2.0 mL hydrogen peroxide (20 mM). The increase in absorbance was determined within 60 s against a reagent without enzyme at 470 nm using a spectrophotometer. The amount of crude enzyme that converts one micromole of hydrogen peroxide in one minute at room temperature equals one unit of enzyme activity [33]. 

#### 2.8.2. Catalase (CAT) Assay 

The CAT activity was assayed [34] by mixing 40 µL of enzyme extract and 9.96 mL phosphate buffer (pH 7.0) containing H_2_O_2_ (0.16 mL of 30% H_2_O_2_ in 100 mL of 50 mM phosphate buffer). CAT activity was determined by measuring the rate of H_2_O_2_ absorbance change in one minute against a buffer blank at 250 nm using a spectrophotometer. One unit of enzyme activity is equivalent to the amount of enzyme that reduced 50% of the hydrogen peroxide in one minute at room temperature. 

### 2.9. Lipid Peroxidation 

The level of lipid peroxidation (malondialdehyde; MDA) was measured according to [35]. A total of 200 mg of fresh flag leaf of wheat plants was ground in 10 mL of 5% trichloroacetic acid (TCA) and centrifuged at 15,000 rpm for 10 min. Then, 2.0 mL of the extract was added to 4.0 mL of 0.5% thiobarbituric acid (Mallinckrodt. Inc., Paris, KY 40361, USA) in 20% TCA, heated in a boiling water bath for a half-hour, immediately cooled, and centrifuged at 10,000 rpm for ten minutes. The reading was noted at 532 and 600 nm using a spectrophotometer. By subtracting the absorption value at 600 nm, the absorption coefficient of 155 nmol cm^−1^ was used to assess the MDA content as nmol g^−1^ FW. 

### 2.10. Protein Profile

The rapid freeze-dried leaf samples (0.2 g) were extracted with 1 mL of protein buffer and kept in the freezer overnight and then vortexed for 15 s and centrifuged at 5000 rpm at 4 °C for 15 min. Then, sodium dodecyl sulfate-polyacrylamide gel electrophoresis (SDS–PAGE) was performed [36]. The molecular weight of the isolated proteins was estimated using standard molecular weight markers (standard protein markers, 11–180 kDa; Sigma, St. Louis, MO, USA). The protein bands were stained with Coomassie Brilliant Blue G-250 (Sigma, St. Louis, MO, USA). 

### 2.11. Statistical Analysis 

The experiment was statistically analyzed as a split-plot design according to [37]. The significant differences were statistically evaluated by Duncan’s test and one-way analysis of variance (ANOVA) using SPSS, version 18.0 (Statistical Package for Social Science, Copyright 2010, Chicago, IL, USA) to discriminate significance (defined as *p* ≤ 0.05). The least significant differences (LSD) at 5% were calculated for means comparisons.

## 3. Results

### 3.1. Growth Parameters

Gamma radiation at 25 Gy significantly decreased flag leaf area, shoot dry weight, and root dry weight while showing no significant difference in plant height, root length, no. of leaves, shoot fresh weight, and root fresh weight. Moreover, except for root fresh weight, gamma radiation at 50 Gy significantly decreased (*p* < 0.05) the shoot and root lengths, no. of leaves, flag leaf area, shoot fresh and dry weights, and root dry weight of wheat plants compared with the control group (Table 1). 

Foliar application of stigmasterol at 100 ppm showed a significant effect on flag leaf area, root length, and root dry weight but showed no changes in plant height, no. of leaves, shoot fresh and dry weights, and root fresh weight of the non-irradiated. Stigmasterol at 200 ppm increased significantly plant height, root length, no. of leaves, shoot dry weight, and root dry weight while showing no changes in flag leaf, shoot fresh weight, and root fresh weight compared to the control plants. 

The treated plants with 25 Gy + stigmasterol at 100 ppm caused significant increases in shoot and root dry weights, decreases in plant height and shoot fresh weight, and no significant effects on root length, flag leaf area, and root fresh weight compared to the corresponding control. Regarding the irradiated plants with 25 Gy, stigmasterol at 200 ppm significantly (*p* < 0.05) improved the shoot length, root length, number of leaves/plant, flag leaf area, shoot fresh and dry weights, and root dry weight over the control values. 

The irradiated plants with 50 Gy and stigmasterol at 100 ppm, except for shoot fresh weight, showed significant increases in the following growth parameters: shoot length, root length, number of leaves/plant, flag leaf area, shoot dry weight, and root fresh and dry weights (28.26%, 21.33%, 21.41%, 36.33%, 52.63%, 59.84%, and 55.56%, respectively) of wheat plants over the corresponding control. Regarding the irradiated plants with 50 Gy, stigmasterol at 200 ppm significantly (*p* < 0.05) improved the shoot length, root length, number of leaves/plant, flag leaf area, fresh and dry weight of shoot, and root by 43.5%, 33.3%, 42.8%, 47.4%, 39.1%, 129.0%, 95.0%, and 66.7%, respectively, over the control values. 

The effect of stigmasterol wheat plant growth depends on the applied concentration. However, stigmasterol at 100 ppm showed no significant effects on some of the physiological attributes compared to control plants. Foliar application of stigmasterol at 200 ppm could improve the growth parameters in non-irradiated and 25 Gy-irradiated plants. Moreover, 200 ppm could counteract the effects of 50 Gy gamma radiation on wheat plant growth. 

### 3.2. Photosynthetic Pigments 

**Figure 1:**
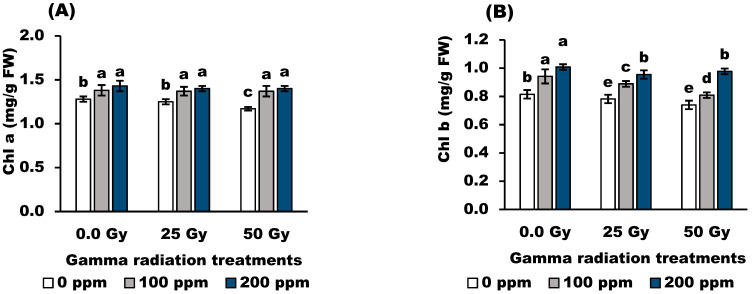

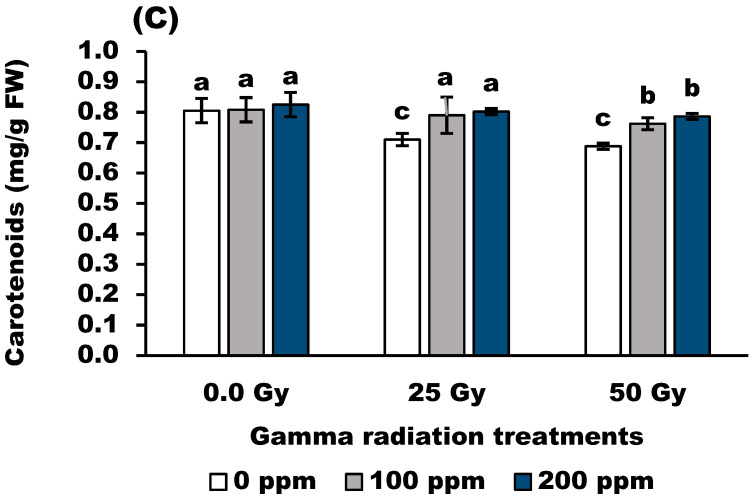
Effect of stigmasterol treatments on photosynthetic pigment; (**A**) Chl *a*, (**B**) Chl *b*, and (**C**) carotenoids contents of wheat plants grown from grain irradiated with gamma rays. The different letters (a–e) show statistical significance at *p* < 0.05; vertical bars indicate ± SD.

Irradiated grains had a significant (*p* < 0.05) influence on the photosynthetic pigments, Chl *a*, Chl *b*, and carotenoids of wheat plants (Figure 1). Radiation at 25 Gy showed no significant effect on Chl *a*, while it decreased significantly Chl *b* and carotenoids compared to the control plants. Radiation at 50 Gy produced progressive damage in the photosynthetic pigments compared to the control plants. On the other hand, the application of stigmasterol at 100 and 200 ppm showed significant (*p* < 0.05) increases in Chl *a* and Chl *b*, and no significant effect in carotenoids in non-irradiated plants compared to the control plants. Moreover, the application of stigmasterol at 100 and 200 ppm significantly (*p* < 0.05) increased Chl *a*-, Chl *b*-, and carotenoid-irradiated plants compared with corresponding control values. Stigmasterol at 200 ppm was the most effective treatment for increasing the photosynthetic pigments. 

### 3.3. Endogenous Phytohormones 

**Figure 2:**
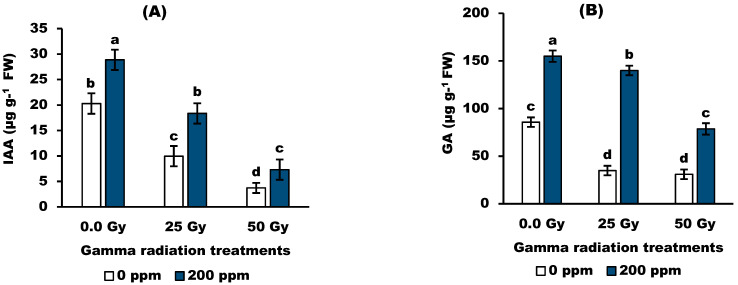

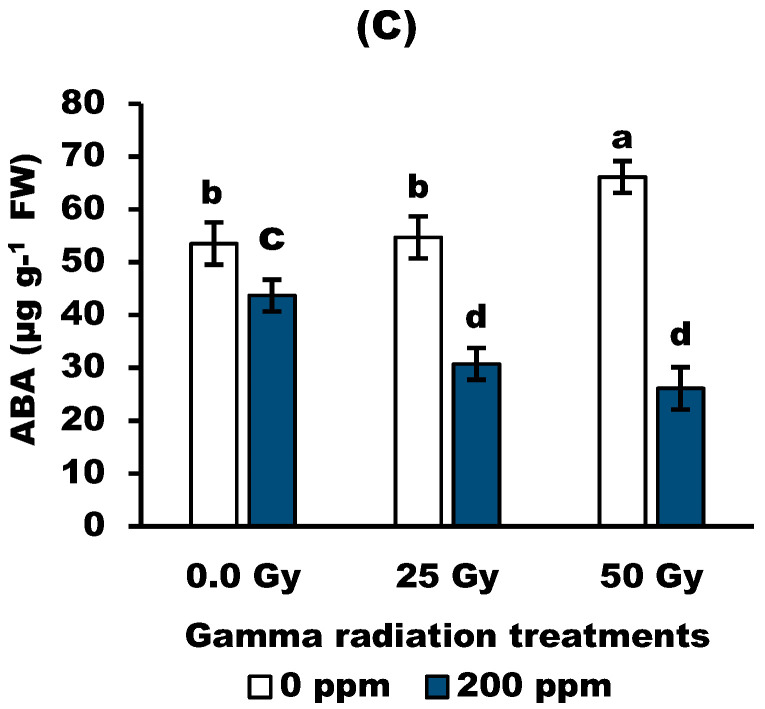
Effect of stigmasterol treatments on endogenous phytohormones; (**A**) IAA, (**B**) GA, and (**C**) ABA of wheat plants grown from grain irradiated with gamma rays. The different letters (a–d) show statistical significance at *p* < 0.05; vertical bars indicate ±SD.

Data in Figure 2 revealed that gamma radiation (25 and 50 Gy) significantly decreased in GA and IAA compared to the control values. Moreover, 25 Gy showed no significant effect on ABA content, while 50 Gy caused a significant increase compared to the control value. In terms of stigmasterol, 200 ppm seemed to be more effective than the control, while 100 ppm treatments were not analyzed. The results showed that stigmasterol at 200 ppm markedly increased the growth promoter (GA3 and IAA) while decreasing the growth inhibitor (ABA) compared with control (non-irradiated plants).

### 3.4. Compatible Solutes

Concerning TSS content, the pretreatments with gamma radiation resulted in dramatic decrements (*p* ˂ 0.05) in total soluble sugar (TSS) in wheat plants. Similarly, TSS decreased with increasing concentrations of stigmasterol in non-irradiated and irradiated plants. The control plants had the highest values of TSS compared to the other treatments.

**Figure 3:**
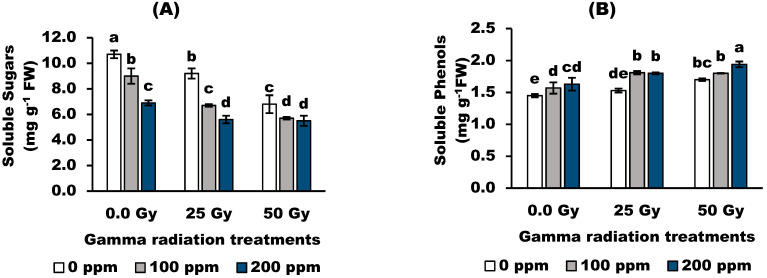

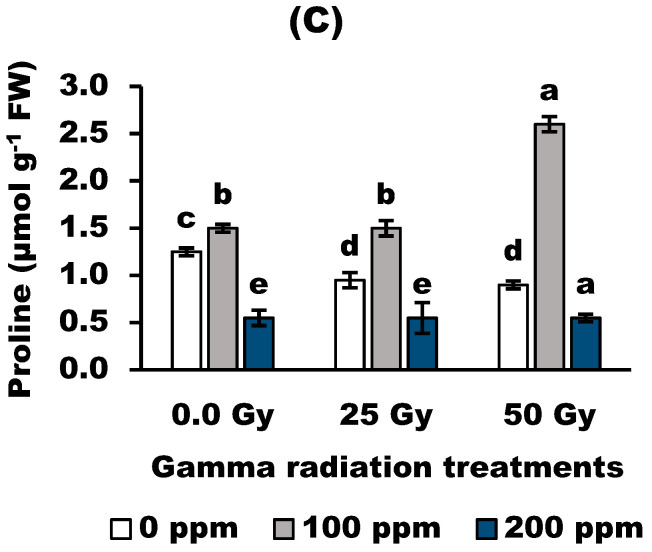
Effect of stigmasterol treatments on compatible solutes; (**A**) soluble sugars, (**B**) soluble phenols, and (**C**) proline of wheat plants grown from grain irradiated with gamma rays. The different letters (a–e) show statistical significance at *p* < 0.05; vertical bars indicate ±SD.

Total soluble phenols showed no significant response to gamma radiation at 25 but were significantly stimulated (*p* < 0.05) with 50 Gy compared with the control value (Figure 3). In addition, stigmasterol at 100 and 200 ppm increased significantly the soluble phenol content in non-irradiated and 25 Gy-irradiated wheat plants over the control values. However, 100 ppm stigmasterol showed no significant effect on total phenols in 50 Gy-irradiated plants compared to the corresponding control. The interaction between 50 Gy and 200 ppm stigmasterol achieved the highest value of total phenols compared to the other treatments.

Gamma radiation dramatically decreased proline content compared to the control value. The effect of foliar application of stigmasterol on the proline content depended on the applied concentration. Stigmasterol at 100 ppm significantly increased the proline content in wheat plants over the corresponding control values. However, the maximum increase (108%) in proline content was in 50 Gy-irradiated plants treated with 100 ppm stigmasterol compared to the control value. Stigmasterol at 200 ppm shifted the proline content to the minimum value below the control value. 

### 3.5. Antioxidant Enzymes

**Figure 4:**
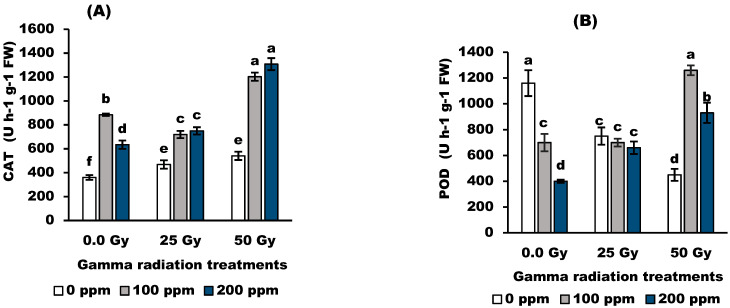

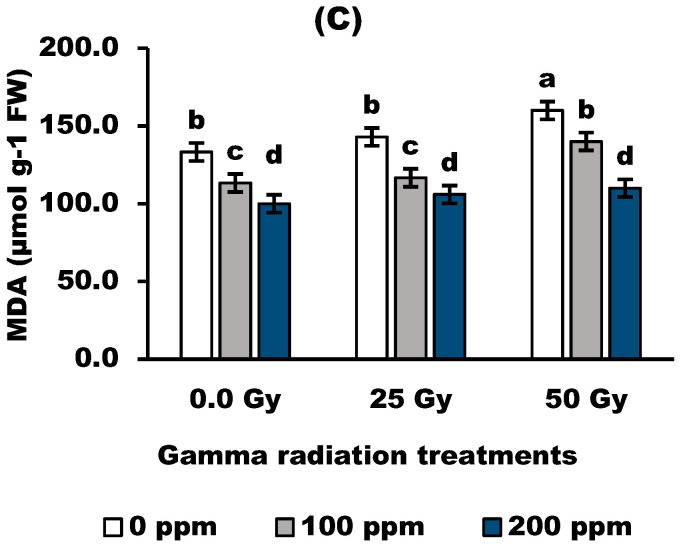
Effect of stigmasterol treatments on the activities of antioxidant enzymes; (**A**) CAT and (**B**) POD, and (**C**) lipid peroxidation of wheat plants grown from grain irradiated with gamma rays. The different letters (a–f) show statistical significance at *p* < 0.05; vertical bars indicate ±SD.

The data in Figure 4 indicated that irradiated grain with gamma radiation at 25 Gy and 50 Gy significantly (*p* < 0.05) increased the activity of the catalase (CAT) enzyme. Stigmasterol at 100 ppm and 200 ppm significantly (*p* < 0.05) increased the activity of CAT enzyme in wheat plants originating from irradiated and un-irradiated grains. Irradiated plants with 50 Gy and treated with 200 ppm stigmasterol increased the CAT activity by 13.0 (U/g) over the control 3.6 (U/g). 

Peroxidase (POX) induced a significant decrease (*p* < 0.05) in response to the tested dose of gamma radiation in wheat plants. Radiation at 25 Gy and 50 Gy significantly decreased POX activity by 25.0% and 50.7%, respectively, compared to control values. On the other hand, stigmasterol increased POX activity compared to untreated wheat plants. Stigmasterol at 100 ppm resulted in the highest increase percentage, reaching 49.73, 84.43, and 173.0% in the 0 Gy-, 25 Gy-, and 50 Gy-irradiated plants, respectively, compared to the corresponding untreated plants.

### 3.6. Lipid Peroxidation

Gamma radiation at 25 Gy showed a non-significant effect on lipid peroxidation MDA), while 50 Gy significantly increased (*p* < 0.05) MDA compared with the control value. Moreover, foliar application of stigmasterol lowered (*p* < 0.05) the MDA content in treated plants below the control. Stigmasterol at 200 ppm decreased MDA by 25%, 25.2%, and 31.25%, respectively, in the control plants and the 25 Gy- and 50 Gy-irradiated plants.

### 3.7. Protein Profile 

**Figure 5:**
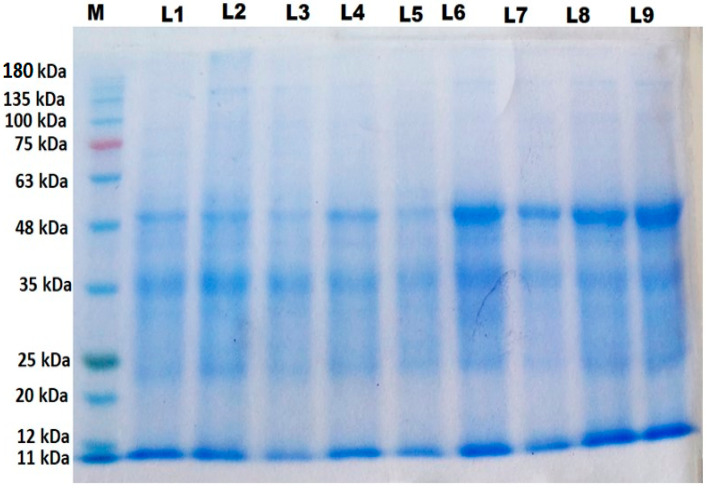
Changes in protein profile in leaves of wheat plants originated from irradiated grains and treated with stigmasterol. L1: Control, L2: 25 Gy, L3: 50 Gy, L4: 100 ppm stigmasterol, L5: 25 Gy + 100 ppm, L6: 25 Gy + 200 ppm, L7: 200 ppm stigmasterol, L8: 50 Gy + 100 ppm, L9: 50 Gy + 200 ppm.

Irradiated grain and application of stigmasterol induced a synthesis or disappearance of different protein bands compared to control plants (Table 2 and Figure 5). There were 23 polypeptide bands ranging from 11 to 185 kDa in the wheat proteins profile. Control plants had six protein bands with molecular weights of 11, 23, 30, 35, 43, and 48 KDa. The results revealed that 25 Gy induced the new polypeptides with 26 and 51 kDa. In addition, gamma radiation at 50 Gy induced polypeptides with 51 and 96 kDa. Moreover, foliar application of 200 ppm stigmasterol caused the appearance of new polypeptide bands with molecular weights of 185, 150, 141, 137, 89, 74, and 37 kDa. 

The combination of 25 Gy and 200 ppm stigmasterol induced the formation of new polymorphic polypeptides with molecular weights of 157, 137, 104, 89, and 79 kDa and unique bands at 137, 105, 89, and 30 KDa. Furthermore, the interaction of 50 Gy and 200 ppm stigmasterol resulted in the appearance of the protein bands at 137, 134, 89, 74, and 38 KDa. The treatment of 50 Gy, stigmasterol, and the interaction (25 Gy and 100 ppm stigmasterol) led to the disappearance of 30 kDa compared to the control plants.

## 4. Discussion

The results indicated no significant difference in plant height, root length, no. of leaves, shoot fresh weight, or root fresh weight between 0 Gy and 25 Gy treatments, while the vegetative growth of wheat plants decreased with radiation dose (50 Gy). The changes in the plant growth exposed to gamma radiation are due to the conversation of metabolic energy and metabolites to ameliorate the oxidative stress effects imposed by ionizing radiation [8]. The growth inhibition of wheat plants may be due to gamma radiation inhibition and the photosynthetic pigments, IAA, GA3, and the antioxidant capacity while inducing ABA and MDA, as mentioned in this study. The negative response of wheat plants is dependent on the nature and extent of the disturbance of cellular metabolism and finally cell damage. For the stigmasterol applied treatments after gamma pretreatment, some of the growth parameters did not differ between 0 ppm and 100 ppm of stigmasterol treatments (Table 1). On the other hand, the application of stigmasterol at 200 ppm improved the growth parameters of both irradiated and un-irradiated wheat plants (Table 1). Stigmasterol plays a vital role in regulating plant growth and development [21,23]. Furthermore, they added that these improvements may be due to increasing the efficiency of water uptake and utilization, enhancing cell division, and/or cell enlargement. 

Leaf chlorophylls are indicators of chloroplast structure and are considered the central part of photosynthetic systems. Data in Figure 1 indicated that Chl *a*, Chl *b*, and carotenoids decreased significantly with 50 Gy gamma radiation doses. These results agree with the previous study [38] on barley plants. The reduction in photosynthetic pigments may be related to the increasing chlorophyll photo-oxidation and damage to the photosynthetic apparatus. Interestingly, the application of stigmasterol can alleviate the damaging effects of gamma radiation on photosynthetic pigment content. The increase in photosynthetic pigment in response to stigmasterol could be attributed to increased photosynthetic apparatus and antioxidant enzyme activities [5]. 

Interestingly, gamma radiation modulates and alters the endogenous hormones of wheat plants represented by the inhibition of GA and IAA, associated with the accumulation of ABA. These results were confirmed by the findings recorded by [39] on barley plants using gamma radiation (50 Gy). Moreover, foliar spraying of the irradiated plants with 200 ppm stigmasterol showed a marked increase in growth promoter levels (GA3, IAA), while the growth inhibitor (ABA) was decreased as compared with control plants. Similarly, the authors in [40] found that 150 or 200 ppm of stigmasterol resulted in the highest GA3 and IAA and the lowest ABA in both cultivars when compared to untreated plants. In addition, the authors in [41,42] stated that stigmasterol increases phytohormones, which can act as messengers and regulators of plant growth and development. The changes in the endogenous hormones were correlated with the changes in the activity of antioxidant enzymes, MDA, and proline, suggesting that the application of stigmasterol plays a central role in counteracting the injurious effects of gamma radiation.

In addition, changes in total soluble sugars in wheat plants may indicate that gamma radiation has induced oxidative stress [43]. The accumulation of TSS by stigmasterol may be due to scavenging ROS to maintain them at the optimum level, hence protecting cell metabolism from free radicals. This explanation was supported by the fact that stigmasterol may act as a photosynthesis activator in wheat plants (Figure 1).

Phenolics are important constituents with scavenging ability due to their hydroxyl groups, which hence may contribute directly to their antioxidant properties. Total phenol content was increased in 50 Gy-irradiated wheat plants. The alterations in the effect of gamma radiation on phenols may occur because irradiation can break the chemical bonds of bioactive compounds, releasing soluble phenolics with low molecular weight and increasing the antioxidant potential of these compounds [44]. On the contrary, stigmasterol increased total phenol content compared to untreated plants. The significant positive correlation between stigmasterol and total phenol content may be induced by protective mechanisms against cell damage resulting from oxidative stress. These findings are consistent with those of [45], who demonstrated that stigmasterol promoted the antioxidant defense mechanism to counteract the negative effects of gamma radiation on faba bean plants.

Regarding proline, gamma radiation leads to a marked decrease in proline content. These results may be due to the radiosensitivity of proline or its oxidation using free radicals generated by gamma irritation. Moreover, stigmasterol stimulated proline content in irradiated and un-irradiated wheat plants. Similar results were obtained by [42] on flax plants. The results may be due to the role of stigmasterol, which stimulates proline content with high antioxidant properties for use in repair mechanisms against radiation effects on plant cell metabolism.

The application of gamma radiation (25 and 50 Gy) decreased the activity of POX enzymes while increasing the CAT activity. Ionizing radiation can trigger the production of ROS by interacting with atoms or molecules in the cell, which is called water radiolysis [38]. The results suggest that the increased activity of CAT induced by gamma irradiation can scavenge excess ROS, especially with the inhibition of peroxidase activity, consequently leading to the enhancement of the antioxidant capacity to overcome oxidative stress induced by water radiolysis. Moreover, treatment with stigmasterol increased POX and CAT activities (Figure 4). It appears that the increase in antioxidant enzymes may be due to the mediated role of stigmasterol in detoxification mechanisms against radio-oxidative stress. 

Lipid peroxidation was markedly increased in the 50 Gy-irradiated wheat plants while decreasing with exogenous stigmasterol treatment. The increase in lipid peroxidation due to gamma radiation treatments was confirmed by [46] on the Black gram (*Vigna mungo* L.), who explained that ROS can react with nearly all cell constituents, which triggers free radical chain reactions that eventually cause membrane lipid peroxidation. They added that the membranes lose their stability, and their permeability is enhanced, leading to damage to the cell structure and disturbances of normal physiological functions as a result of free radical reactions. Moreover, treated wheat plants with stigmasterol improved stress tolerance by decreasing membrane lipid peroxidation in comparison to the corresponding control. This indicates that stigmasterol has a protective role in counteracting the damage induced by gamma radiation, resulting in the induction of antioxidant enzymes (POX and CAT) and non-enzymatic compounds (phenols and proline) associated with lower lipid peroxidation of wheat plants.

Gamma radiation induced the new polypeptides with molecular weights (Table 2 and Figure 5). These results agree with [47] on fenugreek and [38] on barley plants. They indicated that the formation of new bands (unique) is frequently caused by various DNA structural changes (e.g., breaks, transpositions, and deletions), which cause changes in amino acids and, as a result, the protein generated. Furthermore, proteins may play a role in signal transduction, anti-oxidative defense, and osmolyte production, all of which are crucial to the function and growth of plants [48]. In this regard, the authors in [49] suggested that a band with a molecular weight of 51 kDa could be linked to the Rubisco activase enzyme. Ribulose-1, 5-bisphosphate carboxylase activase is a key enzyme that initiates both photosynthetic and photo-respiratory carbon metabolism. Moreover, according to [45], a protein with a molecular weight of 26 KDa appears to be osmotically expressed in flax and sunflower plants under salinity stress to aid survival in stressed environments.

## 5. Conclusions

Gamma radiation at 25 Gy showed no significant difference in some growth parameters, Chl *a*, ABA, soluble phenols, and MDA compared to the control values. Gamma radiation at 50 Gy caused growth inhibition and lowered the photosynthetic pigments, promoting hormone and antioxidant capacity while inducing ABA hormone and lipid peroxidation_._ The foliar application of stigmasterol, especially at 200 ppm, on wheat plants originating from gamma-irradiated grains, improved the photosynthetic pigments, induced an accumulation of osmolytes, phenols, the activity of antioxidant enzymes, and new polypeptides, as well as resynthesized the missed bands by radiation. It is also noteworthy that the stimulatory effects persisted in plants pretreated with stigmasterol throughout, promoting hormones while lowering the ABA hormone. Overall, the results proved the effectiveness of stigmasterol at 200 ppm application in alleviating the adverse effects of radiation stress on wheat plants, as indicated by their protective and stimulatory effects on growth attributes and biochemical constituents.

## Figures and Tables

**Table 1:** Effect of stigmasterol treatments on the growth parameters of wheat plants grown from irradiated grains. The different letters (a–f) show statistical significance at *p* < 0.05. Differences are statistically significant at *p* < 0.05; vertical bars indicate ±SD.

Radiation Dose (Gy)	Stigmasterol (ppm)	Plant Height (cm)	Root Length (cm)	No. of Leaves	Flag Leaf Area (cm^2^)	Shoot Fresh Weight (g)	Shoot Dry Weight (g)	Root Fresh Weight (g)	Root Dry Weight (g)
0	0	60.00 ^b^	10.67 ^ab^	6.00 ^c^	20.10 ^b^	6.08 ^ab^	0.75 ^bc^	1.50 ^cd^	0.17 ^c^
	100	63.00 ^a^	11.00 ^a^	6.00 ^c^	25.77 ^a^	6.12 ^ab^	0.76 ^bc^	1.26 ^de^	0.24 ^b^
	200	68.00 ^a^	11.00 ^a^	8.33 ^a^	22.67 ^ab^	6.21 ^ab^	0.88 ^a^	1.61 ^bc^	0.28 ^a^
25	0	58.67 ^b^	9.67 ^bc^	5.67 ^c^	16.37 ^c^	5.64 ^bc^	0.48 ^e^	1.45 ^cd^	0.12 ^e^
	100	52.00 ^c^	10.00 ^bc^	6.33 ^c^	14.17 ^c^	4.70 ^a^	0.66 ^cd^	1.34 ^cd^	0.16 ^c^
	200	68.00 ^a^	12.00 ^a^	7.33 ^b^	23.90 ^a^	6.70 ^a^	0.83 ^ab^	1.51 ^e^	0.14 ^de^
50	0	46.00 ^d^	7.50 ^d^	4.67 ^d^	15.33 ^c^	4.77 ^d^	0.38 ^f^	1.22 ^de^	0.09 ^f^
	100	59.00 ^b^	9.10 ^c^	5.67 ^c^	20.90 ^b^	5.00 ^cd^	0.58 ^d^	1.95 ^a^	0.14 ^de^
	200	66.00 ^a^	10.00 ^bc^	6.33 ^c^	22.60 ^ab^	6.63 ^a^	0.87 ^a^	1.83 ^ab^	0.15 ^cd^
LSD (0.05) for radiation or stigmasterol		2.388	0.844	0.504	1.611	0.389	0.052	0.192	0.015
LSD 0.05 for the interaction		4.136	1.463	NS	2.791	0.6743	0.091	0.332	0.025

**Table 2:** Changes in protein profile in leaves of wheat plants originated from irradiated grains and treated with stigmasterol. L1: Control, L2: 25 Gy, L3: 50 Gy, L4: 100 ppm stigmasterol, L5: 25 Gy + 100 ppm, L6: 25 Gy + 200 ppm, L7: 200 ppm stigmasterol, L8: 50 Gy + 100 ppm, L9: 50 Gy + 200 ppm.

No.	MW	L1	L2	L3	L4	L5	L6	L7	L8	L9
1	180	-	-	-	-	-	-	+	-	-
2	157	-	-	-	-	-	+	-	-	-
3	150	-	-	-	-	-	-	+	-	-
4	141	-	-	-	-	-	-	+	-	-
5	137	-	-	-	-	-	+	+	+	+
6	134	-	-	-	-	-	-	-	-	+
7	105	-	-	-	-	-	-	-	+	-
8	104	-	-	-	-	-	+	-	-	-
9	96	-	-	+	-	-	-	-	-	-
10	89	-	-	-	-	-	+	+	+	+
11	79	-	-	-	-	-	+	-	-	-
12	74	-	-	-	-	-	-	-	-	+
13	64	-	-	-	-	-	-	+	-	-
14	51	-	+	+	-	-	-	-	-	-
15	48	+	+	+	+	+	+	+	+	+
16	43	+	+	+	+	+	+	+	+	+
17	38	-	-	-	-	-	-	-	+	+
18	37	-	-	-	-	-	-	+	-	-
19	35	+	+	+	+	+	+	+	+	+
20	30	+	+	-	-	-	+	-	+	+
21	26	-	+	-	-	-	-	-	-	-
22	23	+	+	+	+	+	+	+	+	+
23	11	+	+	+	+	+	+	+	+	+
No. of Bands		6	8	8	6	8	13	11	9	12

## Data Availability

The data presented in this study are available in the manuscript.
